# The Beneficial Effects of Cyanobacterial Co-Culture on Plant Growth

**DOI:** 10.3390/life12020223

**Published:** 2022-01-31

**Authors:** Jonas Kollmen, Dorina Strieth

**Affiliations:** Bioprocess Engineering, University of Kaiserslautern, 67663 Kaiserslautern, Germany; strieth@mv.uni-kl.de

**Keywords:** cyanobacteria, plants, co-culture, biofertilizer, nitrogen fixation, secondary metabolites

## Abstract

Cyanobacteria are ubiquitous phototrophic prokaryotes that find a wide range of applications in industry due to their broad product spectrum. In this context, the application of cyanobacteria as biofertilizers and thus as an alternative to artificial fertilizers has emerged in recent decades. The benefit is mostly based on the ability of cyanobacteria to fix elemental nitrogen and make it available to the plants in a usable form. However, the positive effects of co-cultivating plants with cyanobacteria are not limited to the provision of nitrogen. Cyanobacteria produce numerous secondary metabolites that can be useful for plants, for example, they can have growth-promoting effects or increase resistance to plant diseases. The effects of biotic and abiotic stress can as well be reduced by many secondary metabolites. Furthermore, the biofilms formed by the cyanobacteria can lead to improved soil conditions, such as increased water retention capacity. To exchange the substances mentioned, cyanobacteria form symbioses with plants, whereby the strength of the symbiosis depends on both partners, and not every plant can form symbiosis with every cyanobacterium. Not only the plants in symbiosis benefit from the cyanobacteria, but also vice versa. This review summarizes the beneficial effects of cyanobacterial co-cultivation on plants, highlighting the substances exchanged and the strength of cyanobacterial symbioses with plants. A detailed explanation of the mechanism of nitrogen fixation in cyanobacterial heterocysts is given. Finally, a summary of possible applications of co-cultivation in the (agrar-)industry is given.

## 1. Introduction

In 2019, the United Nations estimated the world population could grow to around 9.7 billion people in 2050 [1]. This faces mankind with a great variety of challenges, starting with the increasing demand for nutrition. Already today, modern agriculture depends on the use of nitrogen fertilizers in order to ensure consistently high yields [2]. Traditionally, fertilization is carried out in the form of manure or synthetic fertilizer commonly produced using the Haber–Bosch process. Overall, the amount of reactive nitrogen released worldwide by humans has increased about tenfold since the middle of the 19th century [3]. Nevertheless, about 53% of the reactive nitrogen released by humans originates from the fertilizer industry [4]. The production and input of fertilizers into the environment poses a problem due to the release and formation of environmentally harmful products. These include, for example, the greenhouse gas NO_2_. In addition, around 50% of the applied nitrogen-based fertilizer is actually used by the plants and the remaining 50% causes damage in surface waters through acidification and eutrophication [5,6,7]. Due to an oversupply of nitrogen compared to other nutrients, such as basic cations, the composition of wood and the leaves of plants varies, resulting in a lower resistance to environmental influences. Further, due to the rapid growth of nitrogen-loving plants, slow-growing species are outgrown, leading to a loss of plant diversity [8]. Therefore, ways are being sought to ensure a sustainable supply of nitrogen to plants. One potential possibility is offered by nitrogen-fixing organisms that, in symbiosis with plants, provide only the amount of nitrogen that is actually needed [9,10].

Cyanobacteria are amongst the oldest organisms on earth, with their first appearance dating back to 3.5 billion years ago [11,12]. They are often classified as microalgae, though microalgae are eukaryotic plant cells, while cyanobacteria are phototrophic prokaryotes and are very similar to the subclass of gram-negative prokaryotes due to the structure of their cell walls [13]. Thereby, cyanobacteria have a thicker peptidoglycan layer compared to most of gram-negative bacteria [14]. They show considerable morphological diversity, as they are capable of unicellular or filamentous growth, or they can form colonies [15]. Their occurrence is ubiquitous, i.e., they can survive in the most diverse and extreme habitats such as deserts, hot springs, or polar regions [16]. According to their origin, they are divided into aquatic and terrestrial cyanobacteria [17]. Both terrestrial and aquatic cyanobacteria are capable of forming biofilms, whereby terrestrial cyanobacteria grow surface-associated, air-exposed biofilms. Within these biofilms, terrestrial cyanobacteria live embedded in a matrix of an extracellular polymeric substance (EPS). Besides water, the main components of this EPS are polysaccharides, proteins, lipids, and nucleic acids, although the entire composition has not yet been determined [18]. Among other things, the formation of the EPS enables the cyanobacteria to adhere to surfaces. Furthermore, the EPS serves as a protective layer against desiccation and nutrient deficiency by storing water and nutrients, which is also a reason for the cyanobacteria’s survival in extreme regions [19]. Other advantages include protection against antimicrobial agents and aggregation of cells, resulting in higher cell densities, which in turn results in a higher robustness of the biofilm [20]. Most cyanobacteria are able to grow heterotrophically and phototrophically, as well as mixotrophically [13]. In phototrophic growth, they perform oxygenic photosynthesis, i.e., they use light energy to synthesize high-energy organic compounds (carbohydrates) from low-energy inorganic molecules (CO_2_). In this process, water is split by releasing electrons and oxygen is produced [21]. Like eukaryotic phototrophic organisms, cyanobacteria use specialized reaction centers for photosynthesis, namely photosystem I (PSI) and photosystem II (PSII), which are located in the thylakoid membrane. They enable light-induced electron transport from H_2_O to NADP^+^, whose reduced equivalent NADPH is needed in the formation of carbohydrates [22]. For this purpose, photons are first absorbed at the light-collecting complexes of the photosystems. These photons are in turn transferred to special chlorophyll-a pairs in the reaction centers. The chlorophyll-a pairs are designated P680 (λ = 680 nm) for PSII and P700 (λ = 700 nm) for PSI based on their absorption maxima. In PSII, the oxidation of water to oxygen occurs, and the released electrons are directly supplied to an electron transport chain via the cytochrome b_6_f complex located in the thylakoid membrane toward PSI. The proton gradient created via the electron transport chain is used by ATP synthetase for ATP production, which is required in the Calvin cycle for glucose production and CO_2_ fixation. In PSI, the reduction of NADP^+^ to NADPH is carried out by ferredoxin as an electron transporter [23].

Like all phototrophic organisms, cyanobacteria possess the photopigment chlorophyll-a (Chla). In addition, most contain carotenoids (Car) and phycobilins. The latter are bound to proteins and form with them the so-called phycobiliproteins, which in turn combine to form large, light-collecting protein complexes called phycobilisomes. The most important phycobiliproteins are C-phycocyanin (CPC), allophycocyanin (APC), and phycoerythrin (PE). These additional antenna complexes enable cyanobacteria, in contrast to plants, to absorb light between 500 and 680 nm [24]. Thereby, cyanobacteria are able to adjust their pigment and phycobilisome composition depending on environmental conditions, which is called chromatic adaptation [25]. This enables the cyanobacteria to grow phototrophically even in low-light regions, such as in deep water zones [26].

Furthermore, cyanobacteria are also able to grow heterotrophically in complete darkness. Thereby, the cyanobacteria need an external carbon source in the form of carbohydrates [27]. Energy production can be achieved through various metabolic pathways, which include the oxidative pentose phosphate pathway, glycolysis, and the citrate cycle [28]. If the cyanobacteria are able to assimilate carbon dioxide and metabolize carbohydrates at the same time, this is known as mixotrophic growth [29].

Due to their wide range of products, cyanobacteria offer a variety of possible applications. For example, they have been part of the human diet for decades and are used as a food supplement [30]. Since cyanobacteria produce not only chlorophyll-a, but also other pigments and light-harvesting complexes in the form of carotenoids and phycobiliproteins, they are also suitable as a source of natural dyes [31]. Furthermore, they are used in the production of medical products, as they produce a wide range of antibacterial, antifungal, or antiviral metabolites [31,32,33,34]. Additionally, cyanobacteria have recently come into focus as a source for alternative fuels [35]. Thus, it is possible to obtain methane or crude oil from the biomass by pyrolysis [36], or to use the cyanobacteria directly for the production of hydrogen, for example [37]. Moreover, polyhydroxyalkanoates have been found in cyanobacteria [38]. These have comparable properties to polyethylene and polypropylene and represent a biodegradable alternative to thermoplastics [34]. In addition, many other technically useful products can be obtained from cyanobacteria, such as ethanol, fatty acids, or organic acids [36,37,39]. Finally, cyanobacteria are a promising alternative to artificial fertilizers, as they are able to fix nitrogen from the air and make it available to the plants [40].

This review is intended to provide an overview of the effects of cyanobacteria on plant growth. First, the potential plant-growth-promoting effects based on cyanobacteria are presented, then the direct effects of symbiotic growth are described. Thereupon, possible, as well as already established, applications of co-cultures of plants and cyanobacteria in the (agrar-)industry are summarized.

## 2. Nitrogen Fixation

**Figure 1:**
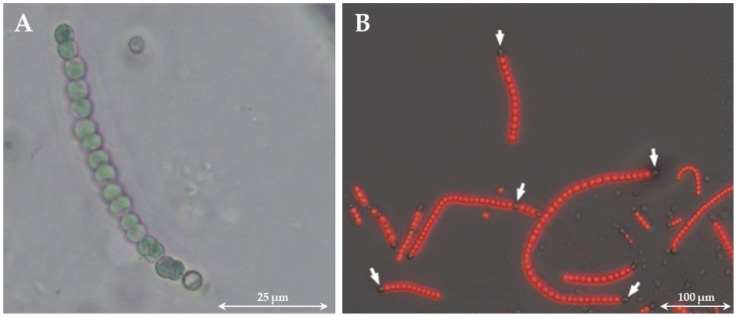
Light microscope image of *Desmonostoc muscorum* after 14 day cultivation in BG11_0_ (without nitrogen) medium (**A**). Overlay of a fluorescence image with a microscopic image of *D. muscorum* (**B**). The red fluorescing cells contain chlorophyll-a and thus an intact photosystem II. The heterocysts do not fluoresce because they lack photosystem II. Some heterocysts are marked by a white arrow. Cultivation conditions: temperature = 30 °C, continuous illumination with 100 µmol_photons_ m^−2^ s^−1^, 120 rpm, and 50 mL BG11_0_ medium in a 300 mL shaking flask.

In the course of their evolution, cyanobacteria have been repeatedly exposed to a wide variety of living conditions, which has led to the fact that they are, nowadays, able to use different sources of nitrogen [41]. These include, for example, ammonium, nitrate, nitrite, urea, or nitrogen-containing amino acids such as glutamine, with ammonium being the preferred source because it is the most reduced inorganic form of nitrogen [41,42,43,44,45,46]. In the absence of a nitrogen source in the medium, diazotrophic cyanobacteria are able to fix atomic nitrogen from the atmosphere and make it biologically available. This is also an important distinguishing feature of cyanobacteria from eukaryotic microalgae, as only cyanobacteria are capable of nitrogen fixation [47]. For this purpose, they need the enzyme nitrogenase, which reduces atomic nitrogen to ammonium under ATP consumption [48,49]. Because nitrogenase is inactivated by oxygen, oxygenic photosynthesis and nitrogen fixation are incompatible processes [50]. For this reason, two different mechanisms have evolved to separate the two processes: (i) temporal separation (day–night rhythm) and (ii) spatial separation (cell differentiation). For example, the cyanobacterium *Cyanothece* sp. strain ATCC 51142 stores glycogen in glycogen granules during the day and fixes nitrogen at night, which is accumulated in the form of a nitrogen-rich polymer (cyanophycin) [51]. In comparison, *Anabaena* sp. PCC 7120 is able to fix nitrogen during the day with the help of specialized cells, the so-called heterocysts [52]. These cells lack PSII and do not perform carbon fixation (the Calvin cycle). This means that no photosynthesis takes place through which nitrogenase cannot be inactivated by oxygen. Heterocysts differ from vegetative cells by their larger and rounder shapes (see Figure 1), a thickening of the cell wall, and an accumulation of cyanophycin granules at the border with neighboring cells [53,54]. Differentiation of vegetative cells into heterocysts is induced by the absence of nitrate and/or ammonium. The time required for the formation of heterocysts depends on the strain, whereby cyanobacteria of the genus *Anabaena* or *Nostoc* usually require between 12 and 20 h for the formation of mature heterocysts [55]. Approximately every tenth cell differentiates into a heterocyst [50]. Unlike vegetative cells, cell division of heterocysts is not possible [56]. Nitrogenase is synthesized within these cells [57]. Due to the thickened cell wall, oxygen penetration is prevented. Additionally, because PSII is inactive in heterocysts, pigmentation decreases [58]. This can be used to distinguish vegetative cells from heterocysts. Vegetative cells contain PSII and thus chlorophyll-a, whose fluorescence can be measured. On the other hand, heterocysts lack PSII and thus chlorophyll-a. Through overlaying a fluorescence image with a microscopic image, the cells can be distinguished (see Figure 1).

**Figure 2:**
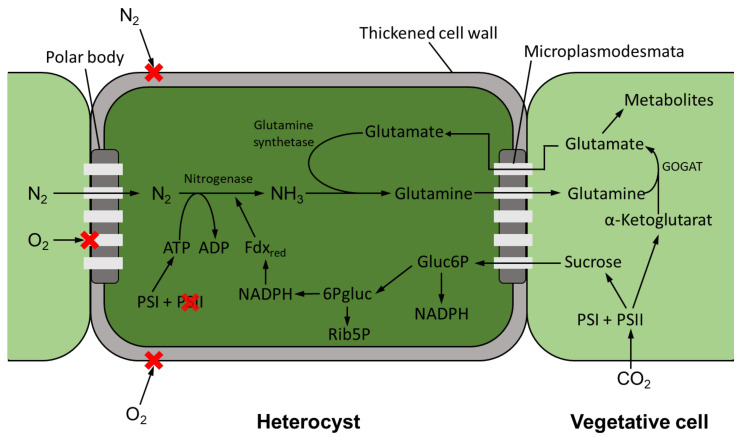
Schematic illustration of nitrogen fixation in heterocysts and metabolic exchange with neighboring vegetative cells. Fdx_red_ = reduced ferredoxin, Gluc6P = glucose-6-phosphate, 6Pgluc = gluconate-6-phosphate, Rib5P = ribulose-5-phosphate, GOGAT = glutamate synthase, PSI = photosystem I, and PSII = photosystem II.

Just like oxygen, nitrogen cannot diffuse directly into a heterocyst via the cell wall and therefore passes from a vegetative cell into a heterocyst via thin cytoplasmic channels (micro-plasmodesmata) (see Figure 2). There, nitrogen is reduced to ammonia or ammonium by the enzyme nitrogenase. While the ATP—necessary to cover the energy demand—is synthesized in the heterocyst by PSI, which remains active, the reducing agent for nitrogen fixation must be provided by neighboring cells [59]. This takes place in the form of twofold sugars, such as sucrose, which are produced in the vegetative cells in which PSII remains active. Sucrose enters the heterocyst via the micro-plasmodesmata, where NADPH is formed as a reducing agent via the pentose phosphate pathway [60]. The reduced nitrogen is transferred to glutamate by the enzyme glutamine synthetase, and thus glutamine is formed, which enters the vegetative cells via the micro-plasmodesmata. Here, the enzyme glutamate synthase is present, which in turn catalyzes the reaction of glutamine with α-ketoglutarate to form two equivalents of glutamate. Glutamate can now re-enter the heterocyst for further synthesis of glutamine or be fed to further metabolic pathways [61]. However, the fixed nitrogen is subsequently not only used by the cyanobacteria to cover their own needs, but can also be released into the medium in the form of ammonium, for example [48].

## 3. Cyanobacterial Metabolites

Plants, however, can benefit from the supply of fixed nitrogen through co-culture with cyanobacteria. For instance, plant-growth-promoting effects have already been demonstrated for various extracts from cyanobacteria [62]. Cyanobacteria are able to synthesize a variety of bioactive molecules with, for example, antimicrobial or growth-promoting properties. These secondary metabolites are often difficult to synthesize chemically due to their complex structure [63]. A classification of the secondary metabolites is possible in different ways: (i) according to their structural classes (see Table 1) and (ii) based on their mode of action on plants or their environment. The latter will also be used in this review, whereby only the beneficial effects of cyanobacterial secondary metabolites are discussed, but it should also be noted that negative effects exist. In this context, the production of cyanotoxins should be mentioned, which have toxic effects on humans and animals and can become a danger, especially with the occurrence of cyanobacterial blooms [64].

### 3.1. Promoting Plant Growth and Growth Condition

The market for growth-promoting substances is currently one of the fastest growing sectors in relation to agriculture, with expected annual growth of approximately 10% until 2027 [97]. Cyanobacteria secrete a variety of potentially growth-promoting secondary metabolites, which, among others, includes auxins. Shariatmadari et al. [98] and Hashtroudi et al. [99] investigated the effects of the water extracts of various cyanobacterial strains on the growth of pumpkin, cucumber, and tomato plants. They were able to show that the extracts contained the auxins indole acetic acid (IAA) and indole butyric acid (IBA). In the application of the extracts, increased values for root length, height, and the wet and dry mass of the plants were observed. Similar results were obtained by Haroun et al. [100] showing that filtrates of the cyanobacteria *Cylindrospermum muscicola* and *Anabaena oryzae* can increase the growth (through increased chlorophyll-a and -b content, photosynthetic activity, and nitrogen and carbon content in leaves) of the plant *Lupinus termis*. In addition to auxins, the cyanobacterial filtrates contained gibberellic acid and cytokinins, which have growth-regulating functions in plants as well (see Section 3). Haroun et al. were able to show that the content of these secondary metabolites increases within the plants through the application of the cyanobacterial filtrates. The content of growth-promoting secondary metabolites depends on the cyanobacterial strain, as shown by Osman et al. [101]. *Nostoc entophytum* contained higher levels of auxins (IAA) and cytokinin, while *Oscillatoria angustissima* had higher levels of gibberellic acid. The different composition also leads to different effects on the growth of pea plants. While fertilization with *N. entophytum* led to higher contents of chlorophyll-a, carotenoids, nitrogen, protein, and exopolysaccharide content compared to *O. angustissima*, higher contents of carbohydrates and phosphate were achieved vice versa.

Cyanobacterial secondary metabolites can not only have a direct effect on plant growth, but also lead to improved growth conditions. Rogers et al. [102] showed that the secretion of polysaccharides by *Nostoc muscorum* led to an increase in soil stability of 18% within 300 days. At the same time, the carbon content in the soil was also increased. Both together led to an increase in the germination rate of lettuce plants by approximately 50%.

### 3.2. Resistance against Plant Disease

Plant diseases can have multiple causes and effects, with the initiators being either living organisms or environmental factors [103]. Especially against diseases triggered by other organisms, cyanobacteria represent a promising solution with their potential antibacterial, antifungal, or pesticidal effects. Shaieb et al. [104] investigated the antibacterial and antifungal effects of fifteen cyanobacteria against seven bacteria and one fungus. Antibacterial and/or antifungal activity was demonstrated for all cyanobacterial extracts, although not every strain is active against every bacterium or fungus. Additionally, the activity depended on the type of solvent (water and ethanol). Many fungi pose a threat to plant growth mainly due to their pathogenic effects. Therefore, Pawar et al. [105] investigated the effects of 40 cyanobacteria against five fungal pathogens. The strength of the inhibitory effect depended on the solvent, whereby methanol extracts with 34.9% inhibition followed by petroleum ether with 30.2% were most effective in inhibiting the pathogens. Chaudhary et al. [106] showed an increased growth of tomato plants suffering from damping off disease by treatment with *Anabaena variabilis*. Treatment with *A. variabilis* even outperformed chemical control by treatment with artificial fungicides (thiram and carbendazim). Similar results were obtained by Dukare et al. [107], who demonstrated the use of cyanobacteria for controlling root disease in tomato plants caused by pathogenic fungi. Kim [108] was able to show that not all cyanobacteria have antifungal effects. A total of 142 cyanobacteria from rice fields were examined, whereby only 6.34% showed an antifungal effect against the seven plant pathogenic fungi tested in this study. This does not automatically mean that the other cyanobacteria have no antifungal effect at all, but it does show that the inhibitory effect is often specialized to a few pathogenic species. The potential range of antifungal activity was investigated by Abo-Shady et al. [109] by testing the activity of extracts of *Anabaena subcylindrica*, *Nostoc muscorum,* and *Oscillatoria angusta* against pathogenic fungi isolated from faba bean roots, stems, and leaves. The fungi belonged to six different families. The inhibitory effect shown by the cyanobacteria against several of the fungi thus indicates that cyanobacteria are generally capable of acting as antifungal agents against a wide variety of genera. However, no cyanobacteria showed inhibition against all fungi, which is why a combination would be favorable. The antibacterial and antifungal effects of cyanobacteria thus represent a promising approach to suppress, or at least minimize, plant diseases caused by pathogenic organisms. Nevertheless, it must be noted that most studies only investigate the in vitro effect of extracts and this does not automatically imply any effect in vivo [110].

Cyanobacterial extracts can not only show antibacterial or antifungal effects, but also pesticidal or herbicidal effects. Biondi et al. [111] observed an antifungal effect against nine fungi, as well as insecticidal (*Helicoverpa armigera*), nematocidal (*Caenorhabditis elegans*), and cytotoxic (*Artemia salina*) effects of methanol extracts of the cyanobacterium *Nostoc* ATCC 53789. While the insecticidal and nematocidal effects are desirable, the application of the strain becomes problematic due to its cytotoxic effect. Furthermore, the herbicidal effect of the strain against grasses could also be shown, although here again the application is complicated by the fact that damage to the roots of tomato plants was also observed. All the effects described were attributed by Biondi et al. to the formation of cryptophycins. In summary, this work shows that cyanobacteria are a promising source of substances for protection against pathogenic eukaryotes, but at the same time, they can also have undesirable effects against useful organisms [111].

### 3.3. Protection against Biotic and Abiotic Stress

Plants are exposed to a variety of environmental influences that have an impact on growth and yield. A distinction can be made between biotic and abiotic stress factors [112]. Cyanobacteria are able to produce a variety of bioactive compounds against biotic stressors, such as bacteria, fungi, or insects. The mortality of tomato plants exposed to biotic stress by Fusarium wilt can be reduced by the use of *Anabaena variabilis*. In this context, the activity of defense enzymes in the tomato plants was also increased [113]. Manjunath et al. [114] investigated the use of *Calothrix elenkii* as a biocontrol agent against biotic stress triggered by the fungi *Phytium aphanidermatum* in the culture of tomato, chili, and brinjal plants, resulting in a decreased mortality and an increased growth of all vegetables. The diseases caused by bacteria and fungi described in Section 3.2 can also be regarded as biotic stress for plants, thus demonstrating the diversity of cyanobacteria as a protective agent against biotic stress. An increase in defense systems combined with an improved growth was observed by Priya et al. [115] for rice plants inoculated with cyanobacteria.

Abiotic stress for plants is very diverse and ranges from weather influences (drought, flooding, or wind) and seasonal influences (temperature or light) to the composition of the soil (salinity, heavy metals, or acidic soil) [116,117]. By using cyanobacteria, the germination of plants can be improved, for example, under drought stress, or in areas with water contaminated with heavy metals or pesticides [118]. For instance, Chua et al. [119] showed that with the help of cyanobacteria, plant colonization and growth are enhanced for the restoration of arid landscapes. Furthermore, Cyanobacteria have developed diverse mechanisms to respond to high soil salinity through the: (i) synthesis and accumulation of protective substances, (ii) maintenance of low ion concentrations within the cells, and (iii) expression of so-called salt stress proteins [120]. Apte et al. [121] observed a change in the protein composition of two cyanobacterial strains, *Anabaena torulosa* and *Anabaena* sp., under salinity effects. The cyanobacteria reacted to salt stress in three different ways regarding their protein synthesis: (i) the expression of some proteins was suppressed, (ii) the expression of some proteins was enhanced, and (iii) through the expression of specialized salt stress proteins. Pandhal et al. [122] observed differences in the protein composition of cyanobacteria *Euthalothece* sp., a halotolerant strain, and *Synechocystis* sp., a moderately halotolerant strain, depending on the salt concentration. While *Euthalothece* showed a stress response at 0% salt, the opposite was the case for 3% and 6% salt. Rodriguez et al. [123] investigated the influence of extracellular products of the cyanobacterium *Scytonema hofmanni* on the growth of rice plants under salt stress. The extracellular products were able to counteract the stress caused by the high salt concentrations. A comparison with the effects triggered by gibberellic acid suggests that *S. hofmanni* produces gibberellin-like plant-growth promoters. Another way to increase the stress sensitivity of plants is through the expression of cyanobacterial flavodoxin within them. This can induce multiple resistances in plants, though de la Pena et al. [124] showed that it can reduce salt stress in the model plant *Medicago truncatula*. The effects of salt stress can also be reduced in bell pepper plants. Bello et al. [125] observed in a soilless cultivation of *Capsicum annum* L. an increase in growth, as well as in the water content of the plants by using a liquid extract of *Roholtiella* sp. Mutale-Joan et al. [126] showed improved growth of tomato plants under salt stress by adding the microalgae *Dunaliella salina* and *Chlorella ellipsoidea* together with the cyanobacteria *Aphanothece* sp. and *Arthrospira maxima*. This improved both the growth of the plants and their composition in terms of chlorophyll content and the content of essential nutrients such as nitrogen, phosphorus, and potassium. The effects of inoculation of high salinity soils go far beyond the synthesis of specific proteins. Various effects have been demonstrated in laboratory and field experiments. Cyanobacteria lead to an increase in nitrogen and carbon content, aggregation status, and water retention, and a decrease in pH, electrical conductivity, exchangeable sodium, and heavy metals, as well as a re-establishment of microbial flora [127,128,129]. Phytohormones, such as salicylic or jasmonic acid, can also contribute to the protection of plants against biotic or abiotic stress by inducing the expression of genes synthesizing for specific proteins [130]. Hussain et al. [131] observed the release of phytohormones from *Nostoc* into the culture media and thereupon a growth stimulation on rice and wheat.

## 4. Symbiotic Association between Plant(-Cell) and Cyanobacteria

Cyanobacteria often grow in symbiosis with various host organisms, such as other prokaryotes [132], eukaryotic protists [133], fungi [134], or plants [135], though only symbiotic associations with the latter will be described in this review. In symbiotic growth, both partners can benefit from each other in different ways. For example, the host organisms (plants) profit from the provision of the nitrogen that the cyanobacteria can fix from the air. This is also the reason why symbiotic cyanobacteria are mostly heterocyst-forming strains [136] and, in particular, belong almost exclusively to the genus *Nostoc* and *Anabaena* [137,138]. The cyanobacteria, called cyanobionts in symbiosis, in turn benefit from the host organisms by the latter providing them with carbon sources, such as sucrose. Here, the cyanobionts can either occur within the host or attach themselves more or less firmly to the host [139]. The strength of the interaction depends on both the cyanobacterium and the plant, whereby the specificity is often such that one eukaryote can only form symbioses with one prokaryote [140]. Gantar et al. [141] observed the colonization of cyanobacteria in different parts of wheat. A large proportion of the cyanobacteria accumulated around the roots in the form of a thick biofilm. However, they also found cyanobacteria in intercellular spaces in the root epidermis and cortex. Single cells were also found within plant cells. Cells associated with the stem or on the surface of leaves were observed as well.

The symbiosis has diverse effects on the growth and development of cyanobionts. For example, an increased heterocyst formation of up to 80% could be observed in the *Gunnera–Nostoc* symbiosis [142]. Thereby, it could also be shown that only about 12% of the fixed nitrogen remains in the cyanobacteria, while the remaining 88% is supplied to the host organism in the form of NH_3_ [142]. The enhanced release of ammonium also causes a decrease in glutamine synthetase activity in the heterocysts. Glutamine synthetase (GS) is the enzyme mainly responsible for the assimilation of ammonium in cyanobacterial heterocysts (see Figure 2). Joseph and Meeks [143] observed a three- to four-fold reduction in GS activity in heterocysts in the *Nostoc–Anthoceros* symbiosis compared to axenic cultivation of cyanobacteria. Reduced GS activity was also shown for the *Azolla–Anabaena* [144] symbiosis and the symbiosis between *Nostoc* and *Anthoceros punctatus* [145]. In symbiosis, the modification of the cyanobionts extends to the point where heterocysts can be formed even in the absence of nitrogen deficiency signs [9]. However, due to the increased heterocyst frequency of cyanobionts, they may be unable to fix sufficient carbon. In these cases, it has been observed that the cyanobionts obtain fixed carbon, for example, in the form of sucrose, from the host plants [137]. Eily et al. [146] investigated the *Azolla–Nostoc* symbiosis. *Nostoc azollae* is not able to survive in nature without the host plant *Azolla*, as the genome of the organism is adapted to the symbiotic way of life. The photosynthetic activity of *N. azollae* is limited, making the organism dependent on a carbon source from its host [147]. Steinberg and Meeks [148] examined the nitrogen fixation rate of cyanobacteria of the genus *Nostoc* with *Anthoceros punctatus* and obtained similar nitrogen fixation rates under dark heterotrophic conditions in symbiosis as achieved by the cyanobacterium exposed to light. This indicates a supply of the cyanobiont by the host with a carbon source. Nevertheless, it is also possible that the cyanobiont transfers both nitrogen and carbon to its host, which occurs, for example, in the symbiosis with bipartite lichens [140]. Apart from obtaining a carbon source, there are few known benefits for the cyanobiont. In general, it probably benefits from a more stable habitat if it accumulates in the host and is also better supplied with additional nutrients, which is partly due to the host’s higher range [136].

It is not only the exchange of nitrogen and carbon that leads to improved plant growth. Another limiting nutrient for plant development is phosphorus [149]. Cyanobacteria are partially capable of converting mineral, insoluble phosphorus, such as ferric phosphate, aluminum phosphate, or hydroxyapatite, into soluble forms that can be used by other organisms [9,149,150]. There are different mechanisms for achieving this, such as the production of organic acids, the synthesis of chelators, a dissimilatory reduction of iron ions, or the enzymatic solubilization of phosphorus compounds. Often, a combination of mechanisms also takes place [151]. Furthermore, plants benefit from a general improvement in the condition of soils and also the provision of growth-promoting substances such as auxins (see Section 3.1). The reduction of biotic and abiotic stress (see Section 3.3) and the protection against plant diseases (see Section 3.2) are also factors that favor the development of symbioses from a plant’s point of view.

Besides the association of cyanobacteria with whole plants, little is known about the formation of symbioses with plant cell cultures. Among the symbioses studied is the co-culture of cyanobacteria with wheat callus. Callus is generally defined as disorganized tissue that plants form in response to stress factors such as injury. In the laboratory, callus tissue is induced by using the plant hormones auxin and cytokinin [152]. Gantar [153] showed that the cyanobacterium *Nostoc* sp. 2S9B in co-culture with wheat callus penetrates the callus and fills the intercellular cavities. The cyanobacteria were enlarged, and the biomass yield increased. At the same time, the production of EPS and the nitrogen fixation rate increased. A penetration of cyanobacteria into callus cells was demonstrated by Gusev et al. [154] using the co-cultivation of *Anabaena variabilis* and tobacco callus. As previously described, symbiosis with plants can lead to increased formation of heterocysts. Gorelova and Kleimenov [155] investigated the effects of co-culturing the cyanobacteria *C. fritschii* and *D. muscorum* with callus cells of the plants *Rauwolfa serpentina* and *Solanum lacinatum* on the nitrogen fixation of the cyanobacteria. They showed that in co-cultivation, the cyanobacteria formed more cyanophycin granules (which serve as nitrogen storage) and formed heterocysts even in nitrogen-containing media. The effects of the symbiosis also depend on the growth status of the plant cells. Thus, these can either stimulate the accumulation of nitrogen in vegetative cells, increase the degradation of nitrogen in them, or initiate the production of heterocysts, even if the cyanobacteria do not detect a nitrogen deficit.

## 5. Potential Application of Artificial Co-Cultures in (Agrar-)Industry

A large part of the nitrogen on Earth exists elementally in the form of gaseous N_2_. Only a few organisms belonging to alphaproteobacterial (e.g., *Rhizobia*), betaproteobacteria (e.g., *Nitrosospira*), gammaproteobacterial (e.g., *Pseudomonas*), firmicutes, and cyanobacteria (e.g., *Nostocales*) have the ability to biologically fix nitrogen from the atmosphere and release it as bioavailable nitrogen such as ammonia [156]. As mentioned before, synthetic fertilizers are mainly used to increase yields in agriculture, and their production as well as their use are harmful to the environment. Nitrogen-fixing cyanobacteria provide a possible solution for a sustainable fertilizer. Since eukaryotic microalgae are not able to fix nitrogen, their application as biofertilizers will not be discussed in detail here, whereby the interested reader is referred to corresponding reviews [47,157,158,159]. Initially, the use of cyanobacteria, especially the heterocyst-forming strains, was mainly limited to fertilizing rice plants [160,161,162,163,164,165]. However, research in the last two decades has shown increased interest and success in fertilizing other crops [166]. Beneficial effects of cyanobacteria have also been demonstrated for the growth of wheat [85,167,168,169,170], tomatoes [113,171,172], maize [173], peas [101,174], and cotton [175]. In each case, the cyanobacteria were added to the soil as a suspension. Farmers can further benefit from such communities in several ways. First, they benefit directly from faster plant growth and thus increased productivity. The production of agricultural products could also become cheaper due to a reduced amount of fertilizer. In addition, cyanobacteria live embedded in a self-produced matrix of EPS that holds the cells together and acts as both nutrient storage [176,177] and a water reservoir [178]. These properties lead to a positive effect on the stability and fertility of the soil [179,180]. In addition to the cells, the EPS also binds and immobilizes soil particles, which counteracts erosion [181]. Further, the EPS improves the water retention of the soil [182]. In addition to its application in soil, the use of cyanobacteria in hydroponic systems should also be mentioned here, as its cultivation in liquid medium facilitates an attachment of the cyanobacteria to the roots of the plants and thus the exchange of nutrients and secondary metabolites. Mutale-Joan et al. [183] give a detailed review on the use of cyanobacteria in hydroponics.

Cyanobacteria are found by nature in rice fields, ensuring that the amount of available nitrogen for the plants is increased [165]. As a result, there is great interest in studying the symbioses between rice plants and cyanobacteria and the specific effects of different symbiotic partners or cultivation conditions on each other. Chittapun et al. [162] investigated the effects of the two *Nostoc* strains, *Nostoc carneum* and *Nostoc commune*, on the growth of rice plants. It was shown that the use of the biofertilizer significantly increased seedling growth and the number of grains per plant compared to the control without fertilizer. A combination of cyanobacteria and chemical fertilizer also had a positive effect [162]. Mishra et al. [161] demonstrated a grain yield increase of up to 19.48% in rice plants co-cultured with cyanobacteria. In hydroponic co-culture, cyanobacteria attach themselves to and within the roots of rice plants, increasing the activity of hydrolytic and defense enzymes in the plant, resulting in increased growth and yield [164]. However, there are not only positive effects. For example, Prieto et al. showed the inhibitory effect of cyanobacterial toxins on the growth of rice plants [184]. Prasanna et al. [173] investigated the growth of maize plants in combination with the cyanobacterial strains *Anabaena* sp., *Anabaena doliolum*, *Nostoc carneum,* and *Nostoc piscinale*. An increase in the amount of pigment in the cyanobacteria was shown in combination with increased growth of the plants. In addition, an increase of the carbon available in the soil by 10–39%, of the nitrogen by 41–43%, and of the phosphate by 13–32% could be demonstrated. The same experiments were carried out by Prasanna et al. [175] with cotton plants. The seed germination rate was increased from 90 ± 2% to 98 ± 5% with the addition of *Anabaena* spp. and *Nostoc* spp. Furthermore, the available amount of nitrogen increased from 97 ± 5 kg ha^−1^ to 190 ± 3 kg ha^−1^, and the yield per plot from 3.6 ± 0.1 kg to 3.8 ± 0.3 kg. Plant weights and heights were also higher under the influence of cyanobacteria. The growth of wheat in symbiosis with cyanobacteria was investigated in several studies. Rana et al. [167] were able to increase the yield and total biomass gain of wheat by co-cultivation with *Anabaena* sp. and *Chalothrix* sp. The same results were obtained by Karthikeyan et al. [85] with the strains *Chalothrix ghosei*, *Hapalosiphon intricatus,* and *Nostoc* sp. Furthermore, the nitrogen and protein content of the harvested grains could be increased [85]. Obreht et al. [168] observed a stimulation of root growth in co-cultivation of wheat with the cyanobacteria *Nostoc* 2S6B, *Nostoc* 2S9B, and *Anabaena* C5 in both nitrogen-containing and nitrogen-free medium. The cyanobacteria attached to the roots and led to increased nitrogen contents in the roots, depending on the cyanobacterial strain. A tight association between cyanobacteria and the roots of wheat was also observed by Fadl-Allah et al. [185] and Gantar et al. [186]. Gantar et al. describe that the strength of the association of the cyanobacteria to the roots is dependent on the strain [186]. Mazhar et al. [169] and Sood et al. [187] also observed increased growth of wheat in co-culture with cyanobacteria. This can be attributed to an increase in the endogenous auxin content in the plants, which in turn correlates with the exogenous auxin production of the cyanobacteria [169]. Signaling substances for the formation of symbioses include the amino acids and sugars secreted by the cyanobacteria [187]. Khollsi et al. [170] also investigated the use of cyanobacteria (*Calothrix* sp. and *Anabaena cylindrica*) to increase the growth of wheat, but in combination with plant-growth-promoting rhizobacteria (PGPR), which resulted in a maximum 36% increase in plant height for *Calothrix* sp. with PGPR. Prasanna et al. [174] reported an up to 39% increased yield of pea plants by co-cultivation of *Anabaena laxa* compared to growth without fertilizer. The protein content of peas was also increased by 11%. Bidyarani et al. [188] conducted similar experiments with the same strain and observed a yield increase of 104% and a 50% increase in the nitrogen content of the plant compared to cultivation without fertilizer. The use of cyanobacteria as biofertilizer for peas has also been studied by Osman et al. [101], resulting in increased germination rates combined with stimulated growth. It was also found that a combination of 50% chemical fertilizer and 50% biofertilizer was most effective, which would still mean a significant reduction in the amount of artificial fertilizer used for one treatment. Suresh et al. [189] investigated the use of two cyanobacterial strains (*Anabaena variabilis* and *Nostoc calcicola*) on the germination rate of five different crop plants (maize, rice, beans, and two types of millet). The results differed greatly between the individual combinations, varying from a germination rate of 5% for *N. calcicola* with beans to 100% for *A. variabilis* with maize, sorghum millet, and beans, with the controls having germination rates of about 50%. They were able to identify indole acetic acid as the dominant auxin in the cyanobacteria [189]. Co-cultivation of tomatoes with cyanobacteria resulted in a lower yield in terms of freshly harvested fruits compared to the use of conventional fertilizers [172]. Kaushik et al. [171] confirmed these observations. In addition, an increase in the nitrogen content of the plant of up to 78% was observed. The quality of the fruit increased when the biofertilizer was used, which could be attributed to an increase in sugar content of up to 33% and an increase in carotenoid content of up to 70%, as well as an increase in the dry matter of the fruit of up to 34% [172]. Shariatmadari et al. [98] used cyanobacterial extracts from the genera *Nostoc* and *Anabaena* to improve the growth of cucumber, pumpkin, and tomato plants. The treatment with the extracts led to an increase in root length and plant height, as well as root fresh and dry weight at the end of cultivation over 40 days. A more than 50% increased germination rate of lettuce plants was achieved by Rogers and Burns [102] using *Nostoc muscorum* as a biofertilizer. Grzesik et al. [190] observed intensified growth and physiological performance of willow plants after biofertilization with *Microcystis aeruginosa*, *Anabaena* sp. and *Chlorella* sp. independent of the application of synthetic fertilizer. The growth of water spinach (*Ipomoea aquatica* L.) was increased by Salamah et al. [191] with the help of the cyanobacterium *Nostoc* sp. SO-A31. In a nitrogen-free environment, the biomass yield, the number of leaves, and the growth of stems and roots could be improved. Rodgers et al. [192] observed an improvement in growth of radish plants co-cultivated with cyanobacteria. While the previously discussed applications of cyanobacteria as fertilizers mainly consider the fertilization of plants via soils, it is also possible to fertilize, for example, by spraying the plant leaves with a solution of cyanobacteria or their extracts [193]. Another field of application for cyanobacteria in the agricultural industry is the treatment of agricultural wastewater. In addition to nutrients such as nitrogen and phosphorus, cyanobacteria can also remove heavy metals, toxins, and pathogens [194]. An overview of the effects of cyanobacterial co-cultures on crops is given in Table 2.

**Figure 3:**
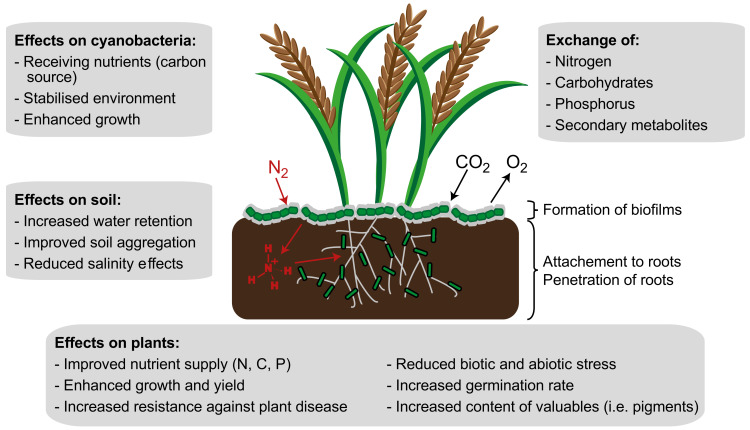
Schematic overview of the effects of a cyanobacteria–plant symbiosis on both partners and their surroundings.

In summary, the suitability of cyanobacteria as biofertilizers has already been proven for many crops. The symbiotic growth not only improves the quantity of many plants, but, in some cases, also the quality. Figure 3 summarizes the positive effects of co-culture on the symbiotic partners and the environment. By mixing commercial fertilizer and cyanobacteria, even better results could be achieved in some cases. Nevertheless, not every cyanobacterial strain formed symbioses with every plant, and an improvement in growth could not be observed for all co-cultivations. Svircev et al. [139], for example, investigated the growth of *Nostoc* 2S9B and *Anabaena* LC2 and C5 during co-cultivations with maize, beet, bean, wheat, and rice plants. The cyanobacteria and plants were brought together in liquid culture. *Nostoc* 2S9B and *Anabaena* LC2 attached to the roots of all organisms, while *Anabaena* C5 attached only to the roots of the wheat plant. Co-cultivation of *Anabaena* C5 with wheat had neither positive nor negative effects on the growth of the organisms. This example shows how different the behavior of cyanobacteria is during co-cultivation with different host plants. Therefore, a prior screening for a suitable strain is necessary for each application. Further, a mixture of cyanobacteria with chemical fertilizer should be carried out in each case to achieve optimal results. It is also conceivable to mix cyanobacteria with other diazotrophic organisms, such as rhizobacteria, which are used, among other things, to fertilize peas [174]. Depending on the plant, the yields and quality of the products can be further maximized in this way.

## 6. Conclusions

Cyanobacteria are a promising source of biological fertilizer. Due to their ability to fix elemental nitrogen from the air and release it into the environment in the form of bioavailable nitrogen, they are able to make this essential element accessible to plants. In addition to nitrogen fixation, plants also benefit from cyanobacterial secondary metabolites. These can protect plants from diseases as well as from biotic or abiotic stress and/or trigger defense reactions in the plants. Cyanobacterial secondary metabolites can furthermore influence plant growth directly or indirectly by improving the growth environment. When cyanobacteria and plants are co-cultivated, symbiotic growth can occur. In this case, the cyanobacteria attach themselves closely to the plant or even penetrate into intra- or intercellular spaces. In most cases, both partners, the cyanobionts and the host, benefit from the symbiosis by exchanging various nutrients. The potential of using cyanobacteria as biofertilizers has already been demonstrated in many studies. The studies focus primarily on agriculturally relevant plants such as rice, wheat, or maize. However, their positive effects on vegetables, such as tomatoes, cucumbers, and other plants, have also been investigated. An improvement in growth, especially in nitrogen-poor environments, could be observed in most cases. Nevertheless, there are also cases of inhibitory effects, or the use of cyanobacteria is often worse in comparison with artificial fertilizer. In the latter cases, however, at least part of the fertilizer can be replaced, which would already be of ecological advantage. In summary, it can be stated that cyanobacteria often have growth-promoting effects on plants, but that further studies are needed for their use in the agriculture industry.

## Figures and Tables

**Table 1:** Cyanobacterial metabolites and their effects on plants.

Class	Metabolites	Cyanobacteria	Effects on Plants	Reference
Phytohormones	auxins, absicic acid, cytokinins, gibberilins, ethylene	*Anabaena* sp., *Anabaenopsis* sp., *Calothrix* sp., *Chlorogloeopsis* sp., *Chroococcidiopsis* sp., *Cylindrospermum*, *Gloeothece*, *Haplosiphon*, *Nostoc* sp., *Oscillatoria* sp., *Phormidium* sp., *Plectonema*, *Rhodospirillum* sp., *Scytonema* sp., *Synechocystis* sp., *Westiellopsis prolifica*	seed germination and growth regulation increased resistance to biotic and abiotic stress expression of genes and synthesis of enzymes nutrient uptake chlorophyll-a, carotenoid, and fatty acid content promoting cell division	[65,66,67,68]
Phenolic compounds	flavonoids, phenolic acids, cell wall phenolics	*Anabaena* sp., *Arthrospira* sp., *Calothrix*, *Chroococcidiopsis*, *Leptolyngbya*, *Nostoc* sp., *Oscillatoria*, *Phormidium*	defense mechanisms color/aroma of flowers and fruit seed germination and growth/development stress reduction flavonoids as unique UV filters signal molecules	[69,70,71,72,73]
Terpenoids	isoprene, limonene, β-phellandrene, linalool, farnesene, bisabolene	*Anabaena* ap., *Synechocystis* sp., *Synechococcus* sp.	increased immunity against disease/toxicity defense mechanisms essential role in the conversion of light into chemical energy assembly and function of photosynthetic reaction centers	[74,75]
Carotenoids	β-, γ-carotene, astaxanthin, canthaxanthin, zeaxanthin, lutein, lycopene, phytoene, echinenone	*Anabaena* sp., *Cylindrospermum* sp., *Microcystis* sp., *Nostoc* sp., *Oscillatoria* sp., *Phormidium* sp., *Synechococcus* sp., *Spirulina* sp., *Tolypothrix* sp.	yellow/orange color of leaves and fruits several aromas in plants essential component in photosynthesis/photoprotection production of phytohormones	[76,77,78,79]
Peptides	peptides, free amino acids, proteins	*Aphanizomenon flos-aquae*, *Calothrix ghosei*, *Cylindrospermum musciola*, *Hapalosiphon intricatus*, *Microcystis aeruginosa*, *Nostoc muscorum*, *Nostoc* sp., *Westiellopsis* sp.	regulation of plant growth and development triggers plant defense responses antioxidative defense systems	[68,80,81,82,83,84,85,86]
Polysaccharides	β-glucans, chitin, lipopolysaccharides, carrageenans	*Arthrospira platensis*, *Nostoc muscorum*, *H. fontinalis*, *P. tenue*	protection against biotic and abiotic stress improved PSII activity improved soil aggregation binding of heavy metals facilitated nutrient uptake	[18,87,88,89,90]
Vitamins	riboflavin, ascorbic acid, thiamine, cobalamine, pyridoxine, nicotinic acid, folic acid, phenothene	*Anabaena* sp., *Chroococcus mimulus*, *Microcystis pulverana*, *Nostoc* sp., *Nostoc muscorum*, *Oscillatoria jasorvensis*, *Phormidium bijugatum*, *Spirulina*	stress reduction improved growth and development increased immunity against disease enzyme cofactors	[91,92,93,94,95,96]

**Table 2:** Investigations into the potential use of cyanobacteria for co-cultivation with crops and their effects on the growth of the organisms.

Plant	Cyanobacterial Strain	Effects	Reference
Cotton	*Anabaena* sp., *Nostoc* sp.	Improvement: germination rate of the seeds yield of cotton plants available N-amount biomass/height of the plant	[175]
Lettuce	*Nostoc muscorum*	Improvement: germination rate	[102]
Maize	*Anabaena* sp., *Anabaena doliolum*, *Nostoc carneum*, *Nostoc piscinale*	Improvement: growth cyanobacteria growth plant C-/P-/N-ratio in the soil	[173]
Peas	*Anabaena laxa*, *Anabaena torulosa*	Improvement: yield protein content in the peas N-content in the plants	[174,188]
Radish	*Anabaena variabilis*, *Nostoc muscorum*	Improvement: growth rate yield	[192]
Rice	*Anabaena laxa*, *Anabaena azollae*, *Calothrix elenkinii*, *Calothrix* sp., *Nostoc carneum*, *Nostoc commune*	Improvement: growth/yield root length amount of seeds per plant activity of hydrolytic and defense enzymes nutrient mobilization plant fitness	[115,161,162,164]
Spinach	*Nostoc* sp.	Improvement: yield number of leaves root length	[191]
Tomatoes	*Anabaena laxa*, *Anabaena variabilis*	Improvement: N-content sugar content carotenoid content CDW tomatoes Reduction: yield CWW tomatoes	[171,172]
Wheat	*Anabaena* sp., *Anabaena* C5, *Chalothrix* sp., *Chalothrix ghosei*, *Hapalosiphon intricatus*, *Nostoc* sp., *Nostoc* PCC 9229, *Nostoc* 2S6B, *Nostoc* 2S9B	Improvement: yield and total biomass (but worse than conventional fertilizer) content of nitrogen and protein in the seeds, roots, and shoots content of chlorophyll in the plants root length endogenous auxin content	[85,167,168,169]
